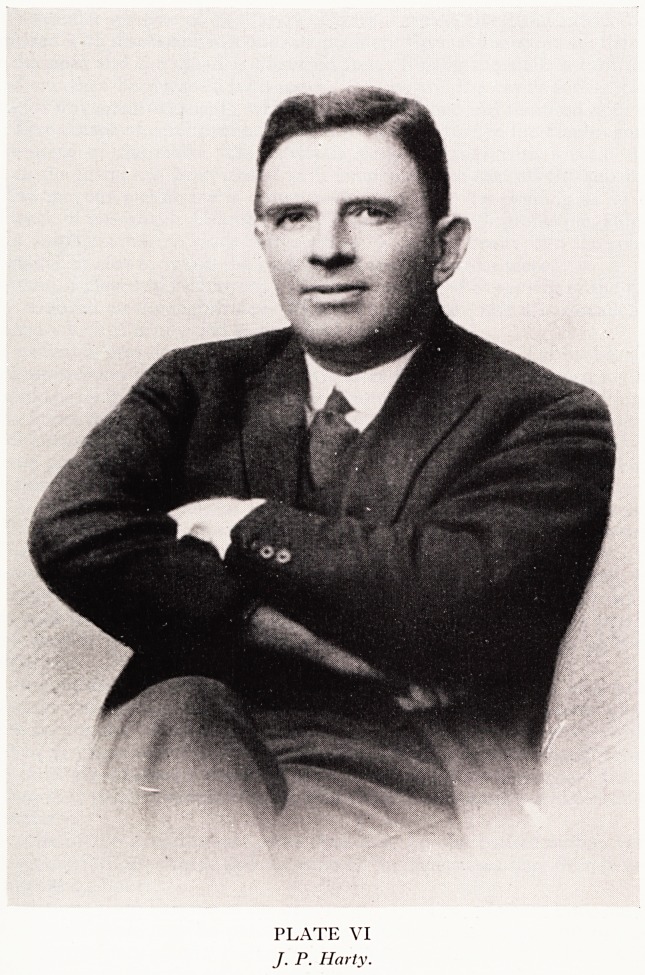# The Rise of Otorhinolaryngology
*Presidential Address to the Medico-Chirurgical Society of Bristol, 12th October, 1961.


**Published:** 1964-04

**Authors:** J. Angell James


### THE RISE OF OTORHINOLARYNGOLOGY*

BY

J. ANGELL JAMES, M.D., F.R.C.S.

I selected this subject at the suggestion of my anaesthetist colleagues because they had observed that the medical profession as a whole is still unaware of the dramatic progress that has been made in otolaryngology in the last thirty years.

To many doctors, as well as to the lay public, E.N.T. surgery has meant the wholesale removal of tonsils and adenoids, the unsuccessful treatment of nasal catarrh, worthless sinus operations, endless prescription of drops for running ears, the dreaded surgery of the mastoid, and quackery or the confession of blank hopelessness in the treatment of deafness.

From the earliest days of specialization the otolaryngologist has been pitied by his colleagues who, not possessing the necessary equipment and skill to examine in detail the dark and obscure recesses of these regions, could not appreciate either the handicaps and difficulty of working under these conditions, or the tremendous nervous tension, concentration, patience, judgement, and width of knowledge required to achieve success in this exacting branch of medicine and surgery.

### THE PAST

The foundation of the speciality of otology was based on the work of the great anatomist Andreas Vesalius of Padua who, in his book published in 1543, described accurately the malleus and incus, and the oval and round windows, but omitted the stapes (which was described later by Ingrassia of Naples). He also described the maxillary, frontal and sphenoidal sinuses. He was succeeded by Fallopius as Professor of Anatomy at Padua (1523–62) who discovered the Fallopian canal, while Eustachius, of tubal fame, held a similar appointment at Rome. Fallopius was also a surgeon and invented the wire snare for removing nasal polypi. The first text-book of Otology, *De Auditus Instrumento*, was written by Volcher Goiter of Groningen, 1535–1600.

In the years that followed, the Italian school continued to provide the most famous names in the sphere of anatomy, medicine, and surgery of the ear, nose, and throat, with Valsalva the first to put treatment on a rational basis and describe auto-inflation of the Eustachian tube. Morgagni (1682–1771), who was the founder of scientific pathology, proved that brain abscess could be due to otitis. Then Cotugno discovered the fluids of the labyrinth, and Scarpa the membraneous labyrinth.

For the surgery of the nasal sinuses, however, we must travel to England, where Nathaniel Highmore (1613–85), practising in Dorset, described in detail the maxillary antrum and gave an account of the diagnosis with a feather probe, and treatment by dental extraction, of a case of suppuration in that sinus.

I cannot pass from this period without referring to tonsillectomy. Richard Wiseman (1622–76), surgeon to Charles II, described the drawing out of the tonsils, ligature of the base and excision with scissors. But it was not until 200 years later that Morell Mackenzie popularized the use of the guillotine for this operation.

The fathers of otology in Great Britain were Yearsley and Toynbee in London and Wilde, whose son was Oscar Wilde, in Ireland. Yearsley was born in Cheltenham in 1805, Toynbee and Wilde were born in 1815. Yearsley founded the Metropolitan Ear Institution and was the first to specialize solely in E.N.T. By 1850 he

* Presidential Address to the Medico-Chirurgical Society of Bristol, 12th October, 1961.

had already done 1,400 tonsillectomies. He is also famous as the founder of the Medical Directory in 1846. Toynbee, although warned that he would make nothing of aural surgery, proceeded to dissect in a few years 2,000 temporal bones which became the Toynbee collection in the Royal College of Surgeons. He killed himself in 1866, just under 100 years ago, experimenting on the relief of tinnitus by inhaling chloroform and prussic acid.

The adenoids were discovered by Meyer of Copenhagen who in 1868, in the search for a cause for deafness with nasal obstruction, palpated the nasopharynx and discovered a mass of tissue.

At the great Allgemienen Krankenhaus in Vienna, Politzer (Plate III) began to specialize in otolaryngology in 1861. He came to England to study under Toynbee, and went to Germany under Helmholtz and to France under Menière. His name and fame as a surgeon, research worker, and teacher is such that he should probably be considered the greatest otologist of the nineteenth century.

In 1873 the first University Aural Clinic was founded in Vienna with Gruber and Politzer as its heads. Each had one room with ten beds in it, Politzer had the female and Gruber the male. This one room served as ward, outpatients, operating theatre and lecture room. From this humble beginning sprang the great Clinic that was the Mecca of E.N.T. surgeons and led the world until the Nazis came to power. Politzer taught in four languages,(and in 46 years 7,000 physicians from all over the world attended his courses). His pupils included Neumann, Alexander and Barany. His dictum should be branded on the heart of every E.N.T. specialist—"Everything is connected with everything".

Strangely enough the Eustachian catheter and the laryngoscope were both discovered by laymen. Guyot, a postmaster of Versailles, in 1724 relieved his own deafness by passing a tube to the back of the nose and injecting fluid. He described this to the Academie Royale des Sciences de Paris. Manoel Garcia, a teacher of singing who described the examination of the larynx with a mirror, should rank as the father of laryngology, for before he introduced this method an accurate knowledge of diagnosis of laryngeal disease was impossible. In September 1854, strolling in Paris in the garden of the Palais Royal, he observed the flashing of the sun in the window panes. He suddenly saw before his eyes the two mirrors of the laryngoscope. He went to a surgical instrument maker and obtained a dental mirror and a hand mirror and began his experiments at home. Garcia himself described what he saw at the International Congress of Medicine in London in 1881.

Another important milestone for the laryngologist was the introduction of cocaine to laryngology in 1884.

The next famous British laryngologist was Morel Mackenzie (1837–92), who was physician at the London Hospital and later founded the Throat Hospital in Golden Square, the centenary of which we are celebrating in 1962. He first published his world-famous textbook *Diseases of the Throat and Nose* in 1880, and founded the *Journal of Laryngology and Otology* in 1887. In 1887 he was called to Berlin to consult on the Emperor Frederick who had cancer of the larynx. He refused to admit this and sanction operation as three biopsies were negative. It was not long before the Emperor succumbed and was succeeded by the Kaiser, who was largely responsible for the first World War.

Sir Felix Semon was the first laryngologist appointed to a General Hospital; this was St. Thomas's in 1882. With the help of Sir Henry Butlin, a general surgeon, he developed the operation of laryngo-fissure.

Nevertheless, so distasteful and unrewarding was work on the ear, nose and throat considered, that in many hospitals otology was delegated to an assistant surgeon and laryngology to an assistant physician. Billroth, the great Viennese general surgeon, who incidentally was the first to perform a laryngectomy for cancer (his patient died and the lesion proved to be tuberculous) said of Otology, "It calls for a certain amount of heroism in a man to sacrifice himself to this therapeutically the most thankless and limited phase of surgery".

It was not until the turn of the century that individuals in many hospitals began to devote their whole energies to these restricted fields, and in many instances it was some years later still before the three branches, laryngology, rhinology and otology, were united into one department. So many of the early specialists were physicians that at first their work was confined to diagnosis and medical treatment. They referred patients requiring major surgery to their general surgical colleagues.

In Bristol, as in many other English teaching hospitals, we were very late in developing the E.N.T. Department. At the Royal Infirmary, Patrick Watson Williams (Plate IV), who was already an Honorary Assistant Physician, was appointed the first Honorary Consultant to the E.N.T. Department in 1906. A. J. Wright (Plate V) was appointed Surgeon to the E.N.T. Department of the General Hospital in 1913, the first mainly surgically trained E.N.T. specialist in Bristol. Both Patrick Watson Williams and John Wright achieved international fame. With Logan Turner in Edinburgh and William Tilley in London, Patrick Watson Williams ranks as one of the fathers of British rhinology. Patrick Watson Williams's work on the suction exploration of the sinuses and the relation of sinus disease to general medicine was read, quoted, and followed in many clinics throughout the world. John Wright is best known for his papers on the importance of focal sepsis in the aetiology of Menière's disease, and for the destruction of the inner ear in this disease by injection of alcohol through the stapes footplate. He delivered the Semon Lecture of the University of London on "The Tonsils" in 1949.

Although Patrick Watson Williams was trained as a physician he soon acquired great surgical skill and designed a number of original instruments, some of which we still use. He was followed by J. P. Harty (Plate VI), a very fine surgeon with a background of experience in general practice from which in eighteen months he passed both primary and final F.R.C.S. England examinations. He was dogged by ill-health following his war service in the first World War. I was his dresser and later house-surgeon, and it was he who fired me with enthusiasm for E.N.T. work and gave me my basic training in it. After his untimely death, which came in 1928, Mr. Eric Watson Williams took over the Department. Mr. Gordon Scarff had been appointed Assistant Surgeon to the Bristol General Hospital shortly before. Thus in 1928 there were four specialist E.N.T. surgeons in Bristol.

We were all trained in general surgery and were able to undertake all major neck as well as ear and nose surgery ourselves. I performed my first total laryngectomy in 1932, the patient living for many years after. Pharyngeal surgery in those pre-sulphonamide and pre-antibiotic days was very arduous, and complicated by sepsis, but with the introduction of the sulphonamides in 1935, sepsis was reduced and much bolder operations could be undertaken.

### PRESENT DEVELOPMENTS

In 1938 came the most dramatic change of all in otological surgery. Julius Lempert, who had studied the pioneer work of Holmgren in Sweden and Sourdille in France, published his description of the first practical one-stage fenestration for the relief of deafness due to otosclerosis.

A new hope had dawned for thousands of patients afflicted with this common and hitherto hopeless disease. As surgeons we had to learn the difficult technique of operating under magnification because of the diminutive scale of the operative field. At first the magnifying loop was used, giving 2½ times magnification. The dissecting microscope was then adapted to human surgery and now gives binocular magnification up to forty times. With technical developments microsurgery has attained a degree of refinement and success that would never have been dreamed of twenty-five years ago.

Every few years a new break-through occurs. In 1955 Rosen described mobilization of the otosclerotic stapes by fracturing the bone fixing the footplate, and thus restoring hearing permanently in a number of patients. We all turned to this in preference to fenestration, as the drum and external auditory canal were left unchanged and the irksome and unpleasant handicap of a large mastoid cavity was avoided. Unfortunately the early promise of results was not fulfilled. Sixty per cent of cases closed again, and it became known as the "frustration" as opposed to the fenestration operation.

Three years later, in 1958, J. Shea of Memphis performed his first total removal of the stapes, replacing the stapes with a polythene tube prosthesis, and sealing the oval window with a vein graft. Many surgeons feared the principle of leaving a foreign body in the middle ear, but untoward reactions have been few. A little later Harold Schuknecht of Detroit discovered by experiments on cats that stainless steel wire was even less irritating than the relatively innocuous polythene, and introduced the fat and stainless steel prosthesis technique. This is the technique that I prefer. In patients without any cochlear defect hearing can be completely restored to normal by these operations. By retaining the transformer effect of the drum and ossicles it is possible to restore a nearly normal relation between air and bone conduction in 90 per cent of patients with otosclerosis.

The operation of stapedectomy by the Schuknecht technique is performed through the external auditory meatus. The posterior half of the drum, with a few millimetres of attached meatal skin, is turned forwards, exposing the bony tympanic ring, the long process of the incus, the stapes, part of the oval window and the round window and promontory. The margins of the tympanic ring are cut away to give wide exposure of the oval window, incus, and stapes. The whole stapes is then removed after dividing the tendon of the stapedius muscle. A $\frac{1}{2}$ mm diamond-and-steel finishing burr is sometimes necessary to remove gross otosclerotic bone. A small piece of fat is then removed from the lobe of the ear and tied securely with a loop of 36-gauge stainless steel wire. A hook is formed in the wire by twisting around a special die, the excess is cut off and the artificial stapes is now inserted so that the fat fits into the oval window tightly and the hook lies around the extremity of the long process of the incus. The loop is then tightened on the incus. Sound waves are now transmitted from the drum through the malleus and incus to the wire, and through the wire to the fat in the oval window, and this produces vibrations in the labyrinthine fluids. The organ of Corti is thus stimulated in the normal way. The tympano-meatal flap is now replaced and retained with a light dressing for a week.

The results are most spectacular in those patients in whom good cochlear function remains. Unfortunately in some otosclerotics there are otosclerotic foci in the modiolus and other parts of the organ of Corti which may interfere with the function of the end-organ itself. This will limit the degree of improvement that can be obtained by freeing or replacing the stapes. In a small proportion of cases also it has been found that as a result of opening the vestibule degeneration of the organ of Corti follows, leading to total loss of hearing. Reports of this in the literature give a percentage varying from 1 to 10 per cent, but McGee of Detroit has performed 300 of these operations without a single case of total cochlear loss following. The results of the more widely practised operation of stapedectomy with polyethylene tube prosthesis and vein graft correspond very closely with those of the wire and fat prosthesis operation, but the tube has been known to be displaced accidentally after complete healing has taken place. It may also happen that if there is a very narrow niche leading down to the oval window, there may not be room for the polyethylene tube to vibrate freely without contact with the walls of the niche, and for this reason it seems likely that many surgeons will prefer to use the wire and fat technique in the future.

The operation of total stapedectomy has almost entirely supplanted that of fenestration of the lateral semicircular canal, but there may still be a place for the latter when, even after complete clearing of the oval window, new otosclerotic bone forms very rapidly and re-closes the window. A fenestration operation in these cases might still be successful, because it is very unusual to find otosclerotic bone in the neighbourhood of the lateral semicircular canal, and the risk of closure is therefore considerably less at this site.

*Ultrasonic Destruction of the Labyrinth*

Ultrasonics were first used in intractable Menière's disease by Arslan in Padua in 1952, to destroy the labyrinth while sparing the cochlea. After I had visited him in 1955 the Royal Hospital obtained an Italian Arslan-Federici apparatus, and with the Medical Physics Department we have worked to overcome the hazards and unreliability of the method. In the Arslan-Federici apparatus the ultrasonic vibrations of 1 million cycles per second are produced by the passage of an alternating electric current of about 3,000 volts through a quartz crystal. The quartz crystal is attached firmly to a metal cone ending in a rod of 5 mm diameter, which in turn is surrounded by a metal tube with an air gap between the two. Although ultrasonic vibrations of this frequency are very freely transmitted by the metal cone and rod, they are not transmitted at all through air. The air gap and metal sheath effectually prevent lateral radiation from the rod. In order to apply the vibrations to the labyrinth it is therefore necessary to apply the end of the metal rod directly to the labyrinth.

The labyrinth is approached through the mastoid process by the usual mastoidectomy route. The operation must be performed under local anaesthesia, so that the reactions of eye nystagmus may be observed. By observation of the nystagmus we are able to know when the labyrinth has been paralysed and the object of our application achieved.

The steps of the operation are as follows: Premedication is given one hour before the operation begins; 50 mg of largactil and 50 mg of pethidine are given. Local anaesthesia is induced by the infiltration of xylocaine and adrenaline over the mastoid process and into the wall of the external auditory meatus. A curved incision is made in the post-auricular fold and carried down through the periosteum over the mastoid process. After reflecting the periosteum, the bone is burred away with electrically driven cutting burrs. The mastoid antrum is opened and the attic exposed forwards sufficiently to show the short process of the incus. The cavity is enlarged, in order to give free access for the application of the tip of the transducer to the lateral semicircular canal. With continuous saline irrigation the bone over the lateral semicircular canal is burred down until a wide blue line is clearly visible, and the surface is flattened so that the tip of the transducer rod may sit as accurately as possible on the bone. We found experimentally that ultrasonics were not transmitted freely by the temporal bone; in fact the half-intensity power distance is only ½ mm at 1 million cycles per second. Fortunately ultrasonic vibrations are transmitted very freely by liquids, and therefore once the vibrations have reached the labyrinthine fluids they are transmitted to all parts of the labyrinth with very little loss of power. The half-intensity power distance in liquids is 15 metres.

Inside the labyrinth the sound vibrations are reflected from the bony surfaces of the canals and vestibule in the same way as audible sound vibrations are reflected from the walls of a speaking tube. Thus nodes and antinodes, where the waves interfere with or supplement the wave motion, produce zones of extremely high and also very low pressures. It is these mechanical effects on the cell walls and contents that are responsible for the damage and ultimate death of the cell.

It is a remarkable thing that the organ of Corti in the human being escapes serious damage in the majority of cases. This is probably because the cochlear duct opens into the vestibule at an oblique angle, and therefore very little of the reflected ultrasonic vibrations are travelling in the direction necessary to enter the cochlea itself. That some of these vibrations enter is, however, quite certain, and we have noted in a considerable number of our patients that there is some damage to the organ of Corti, which is shown by the temporary increase in perceptive deafness that follows immediately after the operation. This usually recovers during the first two or three weeks, but it may take longer, and in 10 per cent of cases a permanent reduction occurs.

After the exposure of the blue line over the lateral semicircular canal the cavity is allowed to fill with saline to act as a coupling and a cooling solution. The treatment rod is now applied over the lateral semicircular canal and the ultrasound is turned on at low intensity. Without cooling, the temperature at the tip of the treatment rod rises very rapidly. Since the facial nerve is only a millimetre or so from the lateral semicircular canal such high temperatures carry a serious risk of causing facial paralysis. In the original Arslan technique no liquid coupling or cooling was employed, and in his hands, and in those of other workers, facial paralysis often occurred. In our own series of fifty-six operations we have had only one case of facial paralysis, and that was due to a failure of the cooling flow which was not observed in time.

The facial nerve is also at risk from the direct application of the ultrasonics if aiming is faulty, or if the beam issuing from the tip of the rod spreads out into side lobes. Using the Schlieren method of rendering the ultrasonic beam visible we found that the development of side lobes occurred when the apparatus was allowed to drift out of tune, as it could do very easily with rising temperature altering the length of the rod in relation to the wavelength. We were able to eliminate this risk by monitoring the tuning of the rod every five minutes during the course of the operation.

Very shortly after the first application of the ultrasound the patient complains of vertigo, which is usually of a rotatory nature, and this is accompanied by nystagmus beating towards the operated side. If the premedication has been too potent, and the patient is very drowsy, the voluntary element of nystagmus may not appear but the deviation of the eyes towards the opposite side, from the irritation of the labyrinth, is easily observed. After some minutes the irritation gradually fades away and is finally replaced by a paralytic nystagmus, away from the operated side, or by a deviation of the eyes towards the operated side.

The enforced period of rest, in order to monitor the apparatus, led us to discover that after a preliminary paralysis the labyrinth could recover and could then again show irritation with nystagmus or deviation, and also with subjective vertigo, nausea and vomiting. We therefore used this as the guide to the dosage necessary to achieve final paralysis, for we continue the application of ultrasound until there is no further irritation visible after a period of rest of approximately fifteen seconds, and then the further application of ultrasound at full power.

At the end of the operation the wound is sutured without drainage.

For the first few days after the operation the patient is very giddy and exhibits a paralytic nystagmus, but this rapidly subsides. Patients are allowed to walk as soon as they are able to balance with assistance. They are usually ready to leave hospital after 12 to 14 days. It is a remarkable fact that after this operation patients seem to suffer very much less disturbance and discomfort than after a surgical labyrinthectomy.

The final degeneration of the labyrinth, with ultimately complete loss of function, seems to take some weeks or months, and after an ultrasonic treatment patients may still have minor attacks of vertigo during the next 3 months.

In addition to our work on the modification of the Arslan-Federici apparatus we have also been designing and testing new types of ultrasonic generators and transducers. The latest Bristol transducer using 3 million vibrations per second, with many novel features, represents an important advance in design. In developing this equipment we have used the sheep for our investigations. We undertook our first human operation with the new equipment one week ago with striking success. In this new equipment the generator has been made to produce an alternating current of 3 million cycles per second which is passed across a lead zirconate titanate crystal of 5 mm diameter held in the tip of the applicator. This equipment is very much more efficient than the Federici apparatus, in which the efficiency was only 2 per cent, whereas in this equipment it is 38 per cent. Very much less heat therefore is generated and the apparatus works at only 100 volts instead of 3,500 volts as used in the Federici apparatus. The crystal is applied directly over the lateral semicircular canal and cooling by liquid conduction is used, in the same manner as when using the Arslan-Federici apparatus.

In spite of its faults the modified Arslan-Federici apparatus has proved very successful in our hands. Not only have a high proportion of patients been relieved completely of their attacks of vertigo, but in 10 per cent of cases the hearing has actually improved, although in 10 per cent of cases also it has been diminished by 10 Db or more. In one case we actually improved the hearing by over 30 Db. Tinnitus also has been relieved in a considerable proportion.

*Transethmosphenoidal Hypophysectomy*

It was Luft and Olivecrona who first suggested and carried out the operation of total hypophysectomy as an alternative to adrenalectomy and oophorectomy for elimination of hormone production in those cases of breast cancer believed to be hormone-dependent. Their approach was by the transcranial route, involving a severe neurosurgical procedure with a high mortality and the risk of damage to the optic and ocular motor nerves and olfactory nerves. Not long after the publication of Olivecrona's work Hamburger of Gothenburg devised an approach through the antrum and ethmoid to the sphenoid, and he has reported a large series of operations with a relatively low mortality and morbidity by this route. For many years the transseptal route has been used for the decompression of tumours of the pituitary, but the distance by this route is long and the field relatively narrow. Chiari in 1911 described the transethmoidal route for decompression of the pituitary.

The application of the operating microscope to the surgery of the posterior sinuses has enabled us to approach the pituitary with safety and precision through a small lateral nasal incision by the Chiari route. Seeing Professor Riskaer perform this operation in Copenhagen three years ago fired my enthusiasm, and after a year of practising and perfecting the instruments required we performed our first operation in March 1960. This was on a patient dying of metastatic breast cancer on whom Mr. Capper found it impossible to perform an adrenalectomy. That patient is still alive and well, and leading a normal life except for her daily dose of cortisone. Since then we have performed over 120 of these operations, and can now in a favourable case complete one in 28 minutes.

The operation is performed under general anaesthesia. A curved incision an inch long is made on the lateral aspect of the right side of the nasal bridge. The periosteum over the nasal bones is divided and the orbital periosteum elevated, carrying with the lachrymal sac, internal tarsal ligament and trochlea of the superior oblique muscle. The anterior ethmoidal artery is divided with diathermy. The ethmoidal cells are then opened until the anterior surface of the right sphenoidal sinus comes into view. The posterior quarter of the nasal septum is then excised to expose the anterior surface

PLATE III

*Politzer.*

PLATE IV
*Patrick Watson Williams.*

PLATE V
*A. J. Wright.*

PLATE VI
*J. P. Harty.*

of the left sphenoidal sinus. The rostrum and the bony anterior wall of the two sphenoidal sinuses are then excised, exposing the anterior surface of the sella turcica bulging into the posterior wall of the sphenoidal sinus. The mucous membrane over this is reflected downwards, and with a dental burr passed through the nose the bone is burred through to expose the dura covering the pituitary gland. The opening is then enlarged with punch forceps until the cavernous sinus on each side, the circular sinus superiorly, and the inferior intercavernous sinus inferiorly are exposed. This leaves an avascular window of two layers of dura covering the gland substance itself. This is incised with diathermy in a cruciate manner. Special dissectors are then passed through the nose and the gland is dissected free. Finally the stalk is torn through. The gland can then be removed with special forceps and the cavity sucked clear and inspected for any small remnants, which are dissected out and removed with cupped forceps. Free escape of cerebrospinal fluid from the aperture in the diaphragma sellae follows removal of the stalk. The cavity is packed tightly with muscle after the aperture in the gland has been closed with aponeurosis to seal off the subarachnoid space. The mucous membrane is drawn back again over the opening as far as possible. A pack of absorbable Calgitex gauze impregnated with proflavine, sulphanilamide, and sulphamethazine made into a cream is packed firmly into the sphenoidal and ethmoidal sinuses to retain the muscle and aponeurosis in place.

The wound is closed in two layers. The pack is allowed to remain in the nose but is usually removed on the eighth day. In order to facilitate this it is threaded with a nylon thread which is firmly tied at the distal end.

As the patient is now deprived of the gland cells producing ACTH his life depends on the administration of cortisone. Directly after the total hypophysectomy there is a very rapid slump in blood pressure unless adequate cortisone has been administered at least 24 hours before the operation. Although it is estimated that the normal production of cortisone only amounts to 25 mg daily we have found it necessary to give our patients 200 mg of cortisone the day before operation, and 300 mg on the day of the operation, owing to the additional stress that the operation entails.

After the operation the patient is maintained under sulphonamide and antibiotic cover with Cristamycin for a fortnight, or longer if there is any leak of cerebrospinal fluid. The operation produces remarkably little general disturbance and no pain, but a proportion of patients develop diabetes insipidus and hypothyroidism, both of which require treatment, the former with pitressin by injection or with snuff, and the latter with thyroxin. Diabetes insipidus dies out usually after three months and causes no further trouble, but the thyroid supplement may have to be continued indefinitely.

After the operation we gradually reduce the cortisone dosage, and as a rule when discharged the patients are down to 75 mg daily in three doses. At a later date it may sometimes be possible to reduce this to 50 mg, and in one or two cases it has been cut down to 35 mgm daily. It is very important that the patients should understand that they must always ensure that they have absorbed the cortisone, and that if they vomit a dose they must have another dose immediately afterwards, or have a dose given intramuscularly.

The results of the operation are often dramatic, for the patient suffers very little shock or discomfort afterwards and the pain of the metastases may be relieved in as short a time as 24 hours.

There are three main hazards to the operation. (1) The sphenoid bone may not be pneumatized and there may be practically no sphenoidal sinus present to guide the surgeon to the pituitary gland. In these cases there is a thick layer of bone which has to be drilled away to find the sella. In doing this the cavernous sinuses and even the internal carotid artery may be damaged. With care and the use of radiographic check during the course of the operation this difficulty can be overcome, but this anatomical condition is found in approximately 5 per cent of cases. So far it has not prevented us from completing any operation, although it has been necessary on two or three occasions to operate in two stages owing to the bleeding from the sinuses during the first stage. This is easily controlled with a muscle pack.

(2) The second risk is that of damage to the cavernous sinuses when they encroach abnormally on the anterior avascular area of the capsule of the pituitary.

(3) The third hazard is the risk of meningitis from ascending infection from the nose. This is most alarming but so far, although we have had this complication following cerebrospinal leak, all have recovered under treatment. When there is a continuing cerebrospinal leak after the operation it may be necessary to re-pack the cavity with muscle, and in every case so far we have been able to arrest the leak.

Up to date we have performed 120 of these operations.

### THE FUTURE

Can we guess in what direction progress will advance? I believe that having mastered to a considerable extent the middle ear we shall now invade the inner ear; already experimental surgery on the cochlea is being carried out in the laboratories, and we hope that our own studies of the inner ear and the labyrinth fluids of the sheep by microchemical methods will in time point the way to new therapy of inner ear diseases and particularly of deafness. In the nose the studies of Malcomson in the neurological control of function, and Miles Taylor of the histo-chemistry, are the beginnings of new understanding. We are only on the fringe of the surgery of the pituitary by the transnasal route.

I am alarmed by the lack of the right type of recruits to train for this work. We have signally failed here in Bristol to attract the junior hospital staff to our department, and most of our registrars in training come from other schools. Nevertheless, I end my address full of hope that as news spreads of the unlimited scope, the immense developments, and the therapeutic triumphs of otolaryngology many of our best graduates will wisely turn to it and share the same thrills of practice and research that we have been privileged to enjoy.

*Acknowledgements*

The work reported has been supported by grants from the United Bristol Hospitals research funds and from the Medical Research Council.

I am deeply indebted to my physicist colleagues Messrs. Freundlich, Bullen and Wells, to my surgical colleagues for referring cases to me, to my Senior Registrar, Mr. G. A. Dalton and my House Officers and Nursing Staff for the large part they have played in developing these methods, and to my colleagues Messrs. Fairman, Freeman and Malcomson for their criticism, help and advice. Also to Professors Messervy and Ottaway and Miss Weaver of the Veterinary Department for their advice and provision of facilities for the animal investigations.

## Figures and Tables

**PLATE III f1:**
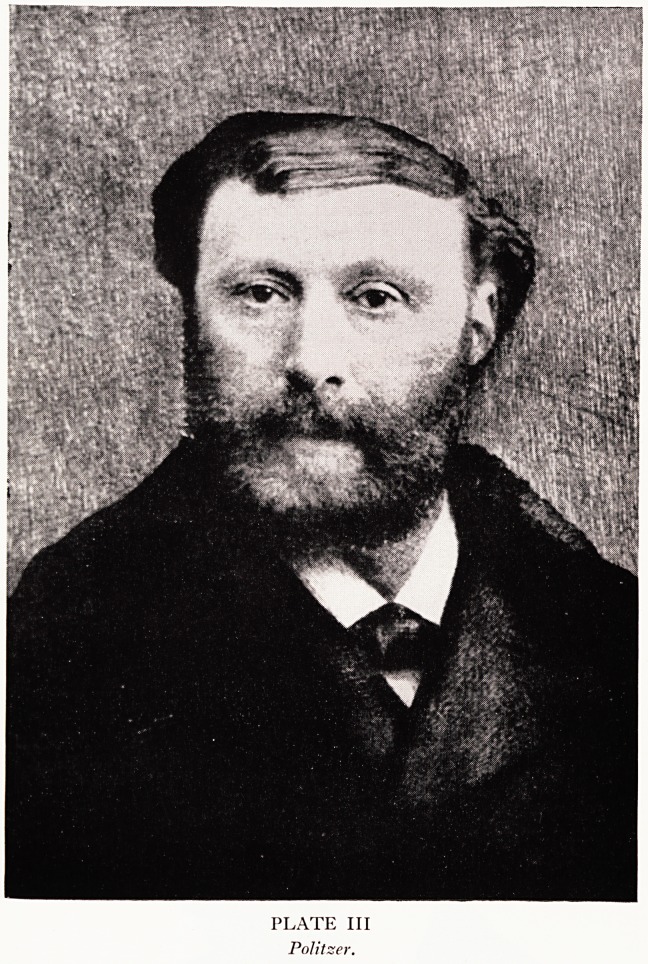


**PLATE IV f2:**
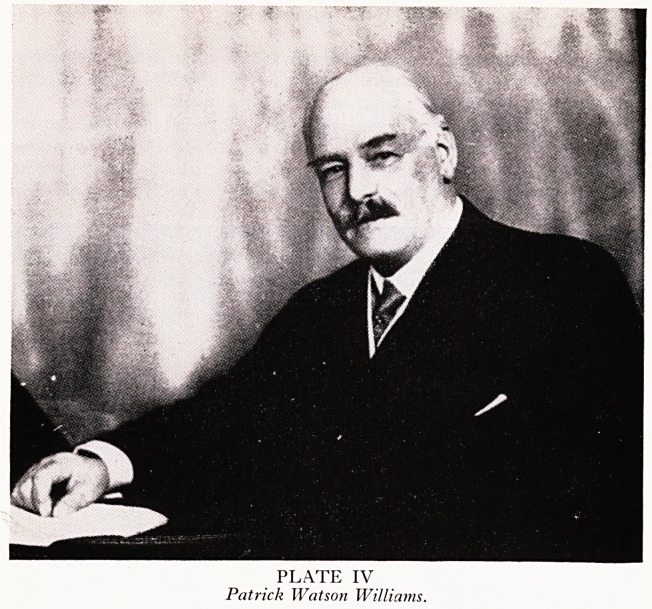


**PLATE V f3:**
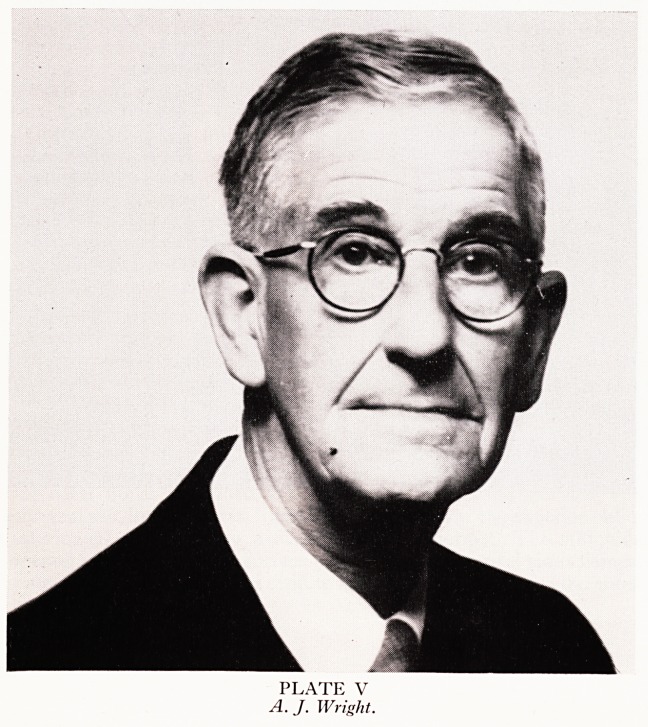


**PLATE VI f4:**